# Adding Salt to Meals as a Risk Factor of Type 2 Diabetes Mellitus: A Case–Control Study

**DOI:** 10.3390/nu9010067

**Published:** 2017-01-13

**Authors:** Lina Radzeviciene, Rytas Ostrauskas

**Affiliations:** Institute of Endocrinology, Medical Academy, Lithuanian University of Health Sciences, Eiveniu 2, 50009 Kaunas, Lithuania; rytas.ostrauskas@lsmuni.lt

**Keywords:** type 2 diabetes, salt, salt intake, risk factors, case–control study

## Abstract

Objective: Type 2 diabetes mellitus (T2DM) is thought to arise from the complex interplay between genetic and environmental factors. It is important to identify modifiable risk factors that may help to reduce the risk of diabetes. Data on salt intake and the risk of type 2 diabetes are limited. The aim of this study was to assess the relationship between adding salt to prepared meals and the risk of type 2 diabetes. Methods: In a case–control study, we included 234 cases, all of whom were patients aged 35–86 years with a newly confirmed diagnosis of T2DM, and 468 controls that were free of the disease. Cases and controls (ratio 1:2) were matched by gender and age (±5 years). A questionnaire was used to collect information on possible risk factors for diabetes. Adding salt to prepared meals was assessed according to: Never, when there was not enough, or almost every time without tasting. The odds ratios (OR), and 95% confidence intervals (CI) for type 2 diabetes was calculated using a conditional logistic regression. Results: The cases had a higher body mass index and a significantly lower education level compared to the controls. Variables such as waist circumference, body mass index, eating speed, smoking, family history of diabetes, arterial hypertension, plasma triglycerides, educational level, occupational status, morning exercise, marital status, daily urine sodium excretion, and daily energy intake were retained in the models as confounders. After adjusting for possible confounders, an approximately two-fold increased risk of type 2 diabetes was determined in subjects who add salt to prepared meals when “it is not enough” or “almost every time without tasting” (1.82; 95% CI 1.19–2.78; *p* = 0.006) compared with never adding salt. Conclusion: Presented data suggest the possible relationship between additional adding of salt to prepared meals and an increased risk of type 2 diabetes.

## 1. Introduction

Diabetes mellitus is presently one of the biggest public health concerns. The emerging pandemic of patients with type 2 diabetes is driven by the combined effects of an aging population, rising levels of obesity, and inactivity [1]. Type 2 diabetes is thought to arise from the complex interplay between genetic and environmental factors, including highly calorific diets [2]. It is important to identify modifiable risk factors for type 2 diabetes mellitus, which may help to reduce the danger of the disease using prevention [3].

About 5000 years ago, the Chinese discovered that salt could be used to preserve foods [4]. Salt then became a large economic importance as it was possible to preserve foods during the winter, and allowed the development of settled communities [4]. Salt was the most taxed and traded commodity in the world, with intake reaching a peak around the 1870s [5]. However, with the invention of the freezer and the refrigerator, salt was no longer required as a preservative [5]. Salt intake had been declining, but with the recent massive increase in the consumption of highly salted processed foods, salt intake is again increasing [6].

The World Health Organization (WHO) suggests a global target of a maximum salt intake of 5 g/day for adults [7]. However, the average salt intake in most countries around the world is approximately 9 to 12 g/day [8]. For Lithuanian adults, the average intake of salt is approximately 8.75 g/day. Even 6.6% of Lithuanian inhabitants always add salt to prepared meals without preliminary tasting [9].

Consensus Action on Salt and Health (CASH), established in the United Kingdom in 1996, actively campaigns to raise awareness on the alleged harmful health effects of salt [10].

In the meantime, scientific literature data on salt intake and the risk of type 2 diabetes mellitus are limited. Therefore, the aim of this study was to assess the relationship between adding salt to prepared meals and the risk of type 2 diabetes mellitus.

## 2. Materials and Methods

### 2.1. Participants and Setting

A case–control study was carried over one year in the region-related to “Dainavos” outpatient clinic of Kaunas, Lithuania. The data source of our study was the inhabitants assigned to the outpatient clinic with primary and secondary level care of 72,000 0–96-year-old inhabitants.

The study included 234 cases, aged between 35 and 86 years, with a newly confirmed diagnosis of type 2 diabetes mellitus, according to the criteria of the World Health Organization, and were tracked over one full year [11]. All patients with type 2 diabetes were studied twice—once after the diagnosis of disease and once before receiving glucose lowering medications.

A total of 468 control subjects from people of the same clinic were recruited. Eligibility criteria were made corresponding to the, gender, age of controls, and a health status free of diabetes and other carbohydrate metabolism disorders. As exclusion criteria for controls, we used history; presence of established diabetes; or impaired glucose tolerance. Hospital staff or students’ relatives were not involved as controls. All controls that had neither impaired glucose tolerance nor diabetes (after an oral 75 gram glucose tolerance test [11] was administered) were individually matched to cases by gender and age (±5 years). During the selection, 42 women and 27 men were recognized as not eligible as controls for the study. The ratio of cases to controls was 1:2.

### 2.2. Ethical Approval

The Kaunas Regional Bioethics Committee approved the design of the study (No. 65/2004). Written informed consent for participation in the study was obtained from all participants.

### 2.3. Study Assessments

Information on age, gender, family history of diabetes, arterial hypertension, plasma triglycerides, daily urine sodium concentration, education, occupational status, household income, marital status, nutrition habits, cigarette smoking, physical activity, and daily energy intake were assessed by a questionnaire designed by our research group. All subjects (cases and controls) were invited to complete the questionnaire themselves. The questionnaire had a good internal consistency of reliability and was applied in the study (Cronbach α (alpha) = 0.824). If there were reasons why someone was not able to complete the questionnaire themselves (e.g., poor general condition, poor eyesight, pathology of upper extremities, or a personal wish for assistance to fill in the questionnaire), interviews were conducted. Interviewers were specially trained and were not made aware of the study’s hypothesis.

Adding salt to a prepared meal was assessed according to the maxims: Never add salt to a prepared meal, add salt to a prepared meal when there is not enough, and add salt to a prepared meal almost every time without tasting.

All participants were asked to fast for 12 h and to avoid smoking as well as heavy physical activity for at least 2 h before examinations. Using WHO guidelines [12], a single investigator took anthropometric measurements. Height was measured without shoes in centimeters (0.1 cm accuracy). Weight was measured in light clothing in kilograms (0.5 kg accuracy). Body mass index (BMI) was calculated as weight (kg)/height (m^2^) [13]. BMI was categorized as 18.5–24.9 kg/m^2^, 25–29.9 kg/m^2^, or ≥30 kg/m^2^ [14]. Waist circumference (WC) was measured by holding non–stretchable measuring tape, snugly around the waist, measured at the midpoint between the bottom rib and the top of the pelvic bones. Hip circumference was measured at the level of the great femur trochanter in centimeters (0.1 cm accuracy) [15]. WC was grouped according to those who were: Less than 80 cm for females and less than 94 cm for males, 80–88 cm for females and 94–102 cm for males, and greater than 88 cm for females and greater than 102 cm for males [16].

Blood pressure was measured with the subject in a sitting position, after at least five minutes of rest, from the left arm using a mercurial sphygmomanometer with a 2.0 mm mercurial column accuracy. Three measurements were taken at intervals of two minutes; the first of these was discarded and blood pressure was obtained from the mean of the latter two measurements. Arterial hypertension was defined as a systolic blood pressure of ≥140 mmHg, a diastolic blood pressure of ≥90 mmHg, and/or current use of antihypertensive medications [17].

Laboratory blood tests included fasting blood samples from subjects’ elbow vein, and venous plasma samples were analyzed for glucose levels. Venous plasma glucose was estimated by the Glucose oxidase phenol 4-amino-antipyrine (GOD–PAP) method (Eppendorf Analyzer, Hamburg, Germany). In order to assess carbohydrate disorders, according to WHO recommendations, 75 g oral glucose tolerance tests were performed and evaluated in the study subjects [11].

Triglycerides were estimated by glycerol phosphate oxidase and peroxidase (GPO–PAP) enzymatic method (Randox analyzer, Antrim, UK).

Participants were given written instructions to collect a 24 h urine sample at home. The urine sodium concentration was determined using the ion selective electrode method (Easy Lyte analyzer, Bedford, USA). The urine sodium concentrations were grouped <mean value and >mean value.

A 24 h dietary recall was used for the assessment of dietary intake. The participants were asked to recall what they had eaten during the previous 24 h. Nutrient values of food were calculated using the Lithuanian Food Composition Tables [18]. The energy intake was grouped <mean value and >mean value.

Occupational status was assessed using the International Standard Classification of Occupations as: Higher education group—1; qualified non-manual work group—2; qualified manual work or less qualified non-manual work group—3; non-qualified manual work or non-qualified non-manual work group—4; pensioner—5; and unemployed or disabled—6 [19].

Levels of education (the number of years) were divided into three categories: Less than 10 years, 11–13 years, and more than 14 years.

Speed of eating was self-reported by the study subjects relative to other subjects with whom they were eating at the same table. Answers used were: Very slowly, relatively slower, the same as other subjects, relatively faster, and very fast. For the question, “How are you eating compared to other subjects?” the categories very slowly and relatively slower compared to other subjects were grouped into one category; relatively faster and very fast were placed in another category.

Smoking was assessed according to habits: Non-smoker, ex-smoker, occasional smoker, and current smoker.

A family history of diabetes was divided into two categories: First-degree relatives with a family history of diabetes and first-degree relatives without a family history of diabetes.

Arterial hypertension was grouped as yes or no.

Plasma triglycerides were grouped as <1.7 mmol/L and ≥1.7 mmol/L.

Morning exercise (duration at least 30 min.) during the last 12 months: No, sometimes, and yes.

Marital status was assessed by grouping into four answers: Married/living together, divorced/separated, single, and widow/widower.

### 2.4. Statistical Analysis

A conditional logistic regression was used to calculate the odds ratio (OR) and corresponding 95% confidence interval (CI) for diabetes mellitus in relation to exposures. Variables such as waist circumference, body mass index, eating speed, smoking, family history of diabetes, arterial hypertension, plasma triglycerides, daily urine sodium concentration, daily energy intake, educational level, occupational status, morning exercise, and marital status were retained in the models as confounders.

All reported trend test significance levels (*p*-values) were two-sided. The χ^2^ test was utilized to calculate the differences between proportions. The level of significance was set at 5%. All the calculations were performed using the standard STATA 7 software program.

## 3. Results

The characteristics of type 2 diabetic patients and of the control group are shown in Table 1. In our study, there were 28.21% men and 71.79% women. The mean age was 64.09 years (SD = 7.85) (range: 39–86 years) for men and 65.23 years (SD = 8.3) (range: 34–86 years) for women. Cases (type 2 diabetic patients) had a significantly lower education level compared to controls. Their body mass index was higher than with controls. There were more controls without a first-degree family history of diabetes than compared with case subjects. The mean daily energy intake among case and control females were 8074.9 ± 1179.3 kJ (mean ± standard deviation) and 8074.5 ± 1177.4 kJ (*p* > 0.05), and among males, correspondingly, 11,777.4 ± 3162.8 kJ vs. 11,706.0 ± 3202.4 kJ (*p* > 0.05). The mean daily urine excretion among cases was not significantly different compared with controls (165.5 ± 50.7 mmol/L vs. 165.2 ± 40.34 mmol/L).

Univariate regression showed that subjects who were adding salt to a prepared meal when there was not enough, or almost every time without tasting, were associated with a higher risk of type 2 diabetes mellitus (crude OR = 1.63; 95% CI 1.19–2.25). A dose-response relationship has been defined between the risk of this disease and adding salt to a prepared meal.

Variables, such as waist circumference, body mass index, eating speed, smoking, family history of diabetes, arterial hypertension, plasma triglycerides, daily urine sodium concentration, daily energy intake, educational level, occupational status, morning exercise, and marital status, were retained in the models as confounders.

Data of unadjusted odds ratios and 95% confidence interval for type 2 diabetes mellitus are presented in Table 2. Data of multivariate logistic regression, showing the relationship between type 2 diabetes mellitus and adding salt to prepare meals, are presented in Table 3. After adjusting for a waist circumference, body mass index, eating speed, smoking, family history of diabetes, arterial hypertension, plasma triglycerides, daily urine sodium concentration, daily energy intake, educational level, occupational status, morning exercise, and marital status, we found an approximately two-fold increased risk of type 2 diabetes mellitus in subjects who add salt to prepared meals when there is not enough or almost every time without tasting (1.82; 95% CI 1.19–2.78; *p* = 0.006) compared with subjects who never add salt to prepared meals.

## 4. Discussion

Our data showed that subjects who add salt to prepared meals when there is not enough, or almost every time without tasting have about a two-fold higher risk of developing type 2 diabetes mellitus compared to subjects who never add salt to prepared meals. The main achievement of our study was the case group that was not affected by glucose lowering treatment. The main weakness is the lack of data about the direct effect of salt on blood glucose levels. However, excess of salt can raise other risk factors for diabetes.

A prospective study in 932 Finnish men and 1003 women, with an average follow-up of 18 years, demonstrated that a higher salt intake (measured by 24 h urinary sodium) was associated with an increased risk of type 2 diabetes independent of potential confounding factors including physical activity, obesity, and hypertension [20]. The mechanism of the association between high intake of sodium and the risk of type 2 diabetes is not well understood.

The WHO recommends for adults to ingest 2.0 g/day of sodium (which corresponds to 5 g of salt/day) based on an assessment of the best available evidence [7]. The primary source of sodium in diet is from salt (NaCl). The terms salt and sodium are often used synonymously, although, on a weight basis, salt is comprised of 40% sodium and 60% chloride; 1 g of sodium is equivalent to 2.55 g of salt; 1 mmol of sodium is equivalent to 23 mg of sodium; and 1 g of salt is equivalent to 17 mmol of sodium [21].

Many observational epidemiological studies conducted worldwide have linked high salt intake and hypertension. The causal relation between habitual dietary salt intake and blood pressure has been established through experimental, epidemiological, migration, and intervention studies [22,23,24]. The first major global study on sodium intake: INTERSALT (10,079 study subjects from 52 centers around the world) demonstrated a significant association with blood pressure, as well as an increase in blood pressure with age. In that study, sodium intake was estimated from 24 h urinary excretion and ranged from 0.2 mmol/24 h (Yanomamo Indians, Brazil) to 242 mmol/24 h (Tianjin, North China) [25]. In the WHO-CARDIAC (World Health Organization Cardiovascular Diseases and Alimentary Comparison) study, performed in 17 countries, 24 h sodium excretion was positively associated with blood pressure among post-menopausal women aged 48–56 years. [26]. The results from epidemiological studies have indicated that subjects with hypertension are more likely to develop type 2 diabetes. The Augsburg surveys, performed between 1984 and 1995, based on 3052 men and 3114 women (aged 35 to 74 years) who participated in one of the three MONICA (Monitoring of Trends and Determinants in Cardiovascular Disease) surveys showed that hypertension was strongly associated with diabetes in men and women [27].

Overweight and obese people usually eat more food than normal-weight people along with a larger quantity of food intake; they also get more sodium. It could be hypothesized that high sodium intake might increase the risk of type 2 diabetes partly through weight gain and hypertension [28,29]. Overweight and obesity are often associated with high blood pressure, and are causally involved in the development of hypertension [16,30,31]. A high salt intake has been suggested as an indirect cause of obesity through the effect it has on soft drink consumption [31]. Salt makes you thirsty and increases the amount of fluid you drink. A reduction in salt intake by 1 g/d was found to be associated with a difference of −100 g/day in total fluid consumption and −27 g/day in sugar-sweetened soft drinks consumption [32]. It has been estimated that a reduction in salt intake from 10 g/day to the WHO recommended level of 5 g/day would reduce fluid consumption by ≈350 mL/day [31]. A study that analyzed the sales of salt and carbonated beverages in the USA between 1985 and 2005 showed a close link between the two, as well as a parallel link with obesity [31].

A randomized community-based intervention trial that was carried out with 550 individuals in Japan demonstrated that dietary counseling for one year reduced salt intake by 2.3 g/day, as measured by 24 h urinary sodium, and this was associated with a decrease of 3.1 mmHg in systolic blood pressure [33]. A reduction in salt from the current average intake of 9–12 g/day to the recommended level of 5–6 g/day will have a major effect on blood pressure and cardiovascular disease; furthermore, it may have other beneficial effects on health [5].

This study has some limitations. The first limitation that needs to be acknowledged and addressed regarding this study is that adding salt to prepared meals was self-reported by subjects, and we cannot exclude reporting bias in the present study. The analyses should be adjusted for the sodium from food and the sodium added during the cooking process. Without this adjustment, the relationship between adding salt to prepared meals and type 2 diabetes remains an hypothesis. Second, the renin-angiotensin-aldosterone system and electrolyte parameters were not studied. Aldosterone plays an important role in glucose metabolism in both animals and humans. The renin-angiotensin-aldosterone system’s deleterious axis increases the production of inflammatory cytokines and raises oxidative stress; consequently, exacerbating insulin resistance and decreasing insulin secretion [34]. Aldosterone impairs insulin receptor signaling by altering the phosphatidylinositol 3-kinase/nitric oxide pathway, by inducing oxidative stress, and by crosstalk between the insulin receptor and the insulin-like growth factor-1 receptor [34,35,36]. The beneficial axis promotes adipogenesis, blocks the production of inflammatory cytokines, and lowers oxidative stress thereby improving insulin sensitivity and secretion [37].

Recently, investigations have demonstrated that excessive salt intake has a pivotal role in the development of inflammatory reactions [38]. Studies have shown that excessive dietary salt intake induced severe inflammatory reactions through augmentation of T-helper 17 cells and their highly inflammatory cytokines [38]. Substantial progress has been made over the last 50 years in studies examining the neural and hormonal basis of thirst and salt appetite [39]. Some of the neurotransmitters and hormones, including the brain area, play a role in water and electrolyte balance [39]. However, further research should be conducted to elucidate the role of high salt intake in the pathophysiology related to disturbances of carbohydrate regulation.

Food can be salted when required for the conservation of food stocks. It is common in countries where season changes reflect significant fluctuations in temperature. Eastern European populations usually consume salted or ready-to-cook meat and fish products, and a great deal of pickled vegetables. Frequent consumption of salted foods may become a habit, which has prompted the development of new taste habits in human mentality. Taste habits become powerful and irresistible. Therefore, even without trying to taste salted food, it, once again, is further salted. This habit can be changed with upbringing and willpower. If people know that excessive salt intake can lead not only to an increase in blood pressure, cardiovascular disease, and renal disease emergence, but also to an increased risk of diabetes. This can become an additional stimulus for a reduction in salt consumption.

## 5. Conclusions

The findings in this study demonstrate a significant relationship between lifestyle and the potential to develop type 2 diabetes mellitus. The presented data has raised the hypothesis of a possible relationship between adding salt to a prepared meal and an increased risk of type 2 diabetes mellitus. This may be related to negative type 2 diabetes-inducing habits.

## Figures and Tables

**Table 1:** Characteristics of cases and controls.

Variable	Category	Cases		Controls		*p*-Value
		*n*	%	*n*	%	for χ^2^
Gender	Males	66	28.21	132	28.21	matched
	Females	168	71.79	336	71.79	
Age (years)	≤44	12	5.13	22	4.70	matched
	45–54	21	8.97	44	9.40	
	55–64	90	38.46	178	38.03	
	≥65	111	47.44	224	47.86	
Family history of diabetes	First-degree relatives without family history of diabetes	166	70.94	422	90.17	
	First-degree relatives with positive family history of diabetes	68	29.06	46	9.83	<0.001
BMI (kg/m^2^)	≤24.9	21	8.97	124	26.50	
	25–29.99	57	24.36	185	39.53	
	≥30	156	66.67	159	33.97	<0.001
Waist circumference (cm)	Females < 80	18	7.69	111	23.72	
	Males < 94					
	Females 80–88	20	8.55	118	25.21	
	Males 94–102					
	Females > 88	196	83.76	239	51.07	<0.001
	Males > 102					
Education (years)	≤10	115	49.15	157	33.55	
	11–13	70	29.91	192	41.03	
	≥14	49	20.94	119	25.43	<0.001
Marital status	Married	137	58.55	296	63.25	
	Divorced/separated	16	6.84	40	8.55	
	Single	13	5.56	25	5.34	
	Widowed	68	29.06	107	22.86	NS
Occupational status	Higher education	16	6.84	46	9.83	
	Qualified non manual work	9	3.85	16	3.42	
	Qualified manual work or less qualified non-manual work	12	5.13	31	6.62	
	Non-qualified manual work or non-qualified non-manual work	7	2.99	28	5.98	
	Pensioner	159	67.95	306	65.38	
	Unemployed or disabled	31	13.42	41	8.76	NS
Eating speed compare to others	Slower	60	25.64	191	40.81	
	The same	44	18.80	103	22.01	
	Faster	130	55.56	174	37.18	<0.001
Adding salt to prepared meal	Never	117	50.00	290	61.97	
	When it is not enough	95	40.60	137	29.27	
	Almost every time without tasting	22	9.40	41	8.76	0.007
Arterial hypertension	No	39	16.67	173	36.97	
	Yes	195	83.33	295	63.03	<0.001
Plasma triglycerides	<1.7 mmol/L	82	35.04	284	60.68	
	≥1.7 mmol/L	152	64.96	184	39.32	<0.001
Energy intake	<mean value	99	42.31	259	55.34	
	≥mean value	135	57.69	209	44.66	NS
24 h Sodium excretion	<mean value	115	49.15	289	61.75	
	≥mean value	179	50.85	179	38.25	NS
Fasting glucose **	<7.0 mmol/L	234				
	≥7.0 mmol/L			468		
Glucose ** after 2 h OGTT	<11.1 mmol/L			468		
	≥11.1 mmol/L	146 *				
Fasting glucose (mean ± SD) **		9.42 ± 3.29 mmol/L		5.88 ± 0.48 mmol/L		<0.001
2 h OGTT glucose (mean ± SD) **		12.17 ± 1.01 mmol/L		8.01 ± 0.64 mmol/L		<0.001

NS: not significant; SD: standard deviation; OGTT: oral glucose tolerance test; * for 88 patients diagnosis of diabetes was made by twice repeating high values of fasting glucose; ** in venous blood plasma.

**Table 2:** Unadjusted odds ratios and 95% confidence interval for type 2 diabetes mellitus.

Confounders	Odds Ratio	(95% CI)	*p*
Adding salt to prepared meal	1.63	1.19–2.25	0.003
Waist circumference	5.02	3.40–7.44	<0.001
Family history of diabetes	3.76	2.48–5.69	<0.001
Arterial hypertension	2.93	1.98–4.33	<0.001
Plasma triglycerides	2.86	2.06–3.97	<0.001
Body mass index	2.61	2.05–3.33	<0.001
Eating speed	1.34	1.20–1.51	<0.001
Smoking	1.24	1.05–1.45	0.009
Occupational status	1.51	1.02–2.22	0.039
Marital status	1.05	0.84–1.31	0.650
Daily energy intake	0.98	0.71–1.34	0.881
Daily urine sodium concentration	0.95	0.56–1.60	0.846
Morning exercises	0.76	0.57–1.02	0.071
Educational level	0.71	0.58–0.88	0.001

**Table 3:** The odds ratios and 95% confidence interval for type 2 diabetes mellitus in relation to adding salt to a prepared meal.

Variable	Category	Cases		Controls		OR^1^ (95% CI)	OR^2^ (95% CI)	OR^3^ (95% CI)
		*n*	%	*n*	%	*p*	*p*	*p*
Adding salt to prepared meal	Never	117	50.00	290	61.97	1.00	1.00	1.00
	When there is not enough or almost every time without tasting	117	50.00	178	38.03	1.68 (1.15–2.46)	1.71 (1.14–2.56)	1.82 (1.19–2.78)
						*p* = 0.007	*p* = 0.009	*p* = 0.006

OR^1^ adjusted for waist circumference, body mass index, and family history of diabetes; OR^2^ adjusted for waist circumference, body mass index, family history of diabetes, daily energy intake, daily urine sodium concentration, eating speed, morning exercise, arterial hypertension, and plasma triglycerides; OR^3^ adjusted for waist circumference, body mass index, family history of diabetes, daily energy intake, daily urine sodium concentration, eating speed, morning exercise, arterial hypertension, plasma triglycerides, smoking, educational level, occupational, and marital statuses.
